# High expression of sperm-associated antigen 5 correlates with poor survival in ovarian cancer

**DOI:** 10.1042/BSR20193087

**Published:** 2020-02-07

**Authors:** Mei Zhang, Ling Sha, Ning Hou, Chuanbing Shi, Lin Tan

**Affiliations:** 1 Department of Pathology, Pukou District Central Hospital, Pukou Branch of Jiangsu Province Hospital, The First Affiliated Hospital of Nanjing Medical University, 166 Shanghe street, Nanjing, Jiangsu, China; 2 Department of Neurology, Affiliated Drum Tower Hospital of Nanjing University Medical School, 321 ZhongShan Road, Nanjing, Jiangsu, China; 3 Department of Pathology, Jiangsu Cancer Hospital, Affiliated Cancer Hospital of Nanjing Medical University, 42 Baiziting, Nanjing, Jiangsu, China; 4 Department of Pathology, Pukou District Central Hospital, Pukou Branch of Jiangsu Province Hospital, The First Affiliated Hospital of Nanjing Medical University, 166 Shanghe street, Nanjing, Jiangsu, China; 5 Department of Obstetrics and Gynaecology, Pukou District Central Hospital, Pukou Branch of Jiangsu Province Hospital, The First Affiliated Hospital of Nanjing Medical University, 166 Shanghe street, Nanjing, Jiangsu, China

**Keywords:** immunohistochemistry, prognosis, SPAG5

## Abstract

Objectives: Sperm-associated antigen 5 (SPAG5), a spindle-binding protein, regulates the process of mitosis. The present study focused on the relationship between SPAG5 expression and the clinicopathological characteristics and prognosis of ovarian cancer. Methods: First, we used the Gene Expression Omnibus (GEO) database to analyze SPAG5 expression in ovarian cancer and its clinical relevance. Subsequently, qPCR test was used to detect SPAG5 mRNA expression in 20 cases of ovarian cancer. The expression of *SPAG5* protein in a tissue microarray containing 102 cases of ovarian cancer was detected by immunohistochemistry. Cox regression and Kaplan–Meier survival analyses were performed to identify the prognostic factors for the 102 ovarian cancer patients. Results: In the GEO datasets, *SPAG5* mRNA expression was significantly higher in ovarian cancer tissues than that in normal ovarian tissues (P < 0.001). qPCR and immunohistochemistry showed that SPAG5 expression in ovarian cancer tissues was significantly higher than that in paracancerous tissues (*P* = 0.002, *P* < 0.001). The high expression of SPAG5 in ovarian cancer was correlated with histological type (*P* = 0.009), lymph node metastasis (*P* = 0.001), distant metastasis (*P* = 0.001), TNM stage (*P* = 0.001), and prognosis (*P* = 0.001). The Kaplan–Meier curve indicated that rates of disease-free survival (DFS) and overall survival (OS) were even lower in patients with high SPAG5 expression. Multivariate analysis showed that SPAG5 expression (*P* = 0.001) and TNM staging (*P* = 0.002) were independent prognostic factors for the DFS of ovarian cancer. Conclusions: These results suggest that high SPAG5 expression was correlated with multiple clinicopathological features of ovarian cancer and can be used as an evaluation indicator for a poor ovarian cancer prognosis.

## Background

Ovarian cancer is a common gynecological malignant tumor, with 295,414 newly diagnosed cases and 184,799 deaths every year worldwide [1]. In China, the incidence and mortality of ovarian cancer rank 8th and 10th among female malignancies, respectively [2]. The numbers of newly diagnosed cases of and deaths due to ovarian cancer in China each year are 52,100 and 22,500, respectively [3]. The histopathological type of approximately 90% of ovarian cancers is epithelial malignant tumor, including serous carcinoma, mucinous carcinoma, uterine endometrial carcinoma, and clear cell adenocarcinoma. Among all gynecological tumors, ovarian cancer has characteristics of late diagnosis, high mortality rate, and the worst prognosis. Approximately 70% of ovarian cancers have already developed into advanced cancer at the time of diagnosis. Although surgical treatment, chemotherapy, radiotherapy, and targeted therapy have developed rapidly in recent years, the 5-year survival of ovarian cancer is only 20% [4–6]. Although tumor markers such as cancer antigen 125 (CA125) and human epididymis protein 4 (HE4) play important roles in the diagnosis and treatment of ovarian cancer, they are still not enough for the individualized and precise treatment of ovarian cancer. Thus, finding markers for early relapse and poor prognosis of ovarian cancer is very important [7–9].

Sperm-associated antigen 5 (SPAG5), which is also known as astrin or hMAP126, is located on chromosome Ch17q11.2 and belongs to the family of spindle-binding proteins. During the middle phase of mitosis, SPAG5 is concentrated at the centromere to regulate the time of spindle assembly and separation of sister chromosomes during mitosis [10,11]. During mitosis, SPAG5 and other related proteins together regulate the activity of kinetochore microtubules and promote the progression of mitosis [12]. Yang et al. [13] found that SPAG5 can inhibit apoptosis and promote cell proliferation. Recent studies have found that SPAG5 is associated with tumor occurrence, development, invasion, and metastasis. Yuan et al. [14] found that SPAG5 was highly expressed in cervical cancer tissues and was associated with poor cervical cancer prognosis and treatment sensitivity to paclitaxel. High SPAG5 expression is a poor prognostic factor for breast cancer and is associated with sensitivity to breast cancer chemotherapy [15,16]. Liu et al. [17] found that SPAG5 can inhibit apoptosis in urothelial carcinoma of the bladder and promote tumor proliferation, which was associated with poor tumor prognosis. Zhou et al. [18] found that SPAG5 was highly expressed in hepatocellular carcinoma (HCC), which suggested poor HCC prognosis. These results indicate that *SPAG5* is an important oncogene involved in the development and progression of tumors and affects the malignant biological behaviors of tumors. However, the prognostic value of SPAG5 expression in ovarian cancer has rarely been studied.

The present study aimed to detect *SPAG5* messenger RNA (mRNA) expression levels in fresh ovarian cancer samples via one-step quantitative reverse transcription-polymerase chain reaction (qPCR), detect SPAG5 protein expression by Western blot (WB), and analyze the SPAG5 protein in an ovarian cancer tissue microarray (TMA) using immunohistochemistry (IHC). The correlation between SPAG5 expression and the clinicopathological characteristics and prognosis of ovarian cancer patients was investigated.

## Materials and methods

### Gene Expression Omnibus (GEO) data collection and mining

Genechip data were downloaded from the GEO database. Specifically, the GSE44104 (submission date: February 6, 2013), GSE12172 (submission date: July 18, 2008), GSE14407 (submission date: January 13, 2009), and GSE40595 (submission date: September 4, 2012) datasets were based on the GPL570 platform (Affymetrix Human Genome U133 Plus 2.0 Array, Affymetrix Inc., Santa Clara, CA). The GSE26712 dataset (submission date: January 19, 2011) was based on the GPL96 platform (Affymetrix Human Genome U133A Array, Affymetrix Inc., Santa Clara, CA). The original expression profile of carboxyl ester lipase (CEL) was subjected to robust multiarray average (RMA) normalization using the Affy package in R language. The mRNA expression level of SPAG5 was further subjected to log2 transformation. The expression levels of genes with multiple expression data are represented by the corresponding mean expression levels. SPAG5 expression in ovarian cancer was divided into a high expression group and a low expression group using the median as the cutoff value.

### Ovarian cancer tissue specimens

A total of 102 ovarian cancer tissue specimens and paracancerous tissues were collected at the Department of Pathology, Jiangsu Cancer Hospital between 2005 and 2015. All specimens were fixed using formalin and embedded in paraffin. All specimens were reviewed by two senior pathologists. Clinicopathological patient information was collected, including gender, age, tumor size and location, histological type, degree of differentiation, and lymph node and distant metastasis. According to the 7th edition of the American Joint Committee on Cancer (AJCC) staging manual, none of the patients received preoperative radiochemotherapy or immunotherapy. The present study was approved by the Institutional Review Board of Pukou Central Hospital, Nanjing, Jiangsu, and carried out in accordance with the World Medical Association Declaration of Helsinki (2019-PK-H052). All subjects provided written informed consent.

### Detection by qPCR

Fresh specimens from tumor and paracancerous tissues from 20 of the above 102 ovarian cancer cases were collected. Methods for RNA extraction, quality control, and qPCR detection were described previously [19,20]. The following primers were used for qPCR: (1) SPAG5, 5′-TGCCCAAACCACCCCGTCAT-3′ (forward) and 5′- TCAGGACTGCCCCATTGCTC-3′ (reverse) and (2) GAPDH, 5′-TGCACCACCAACTGCTTAGC-3′ (forward) and 3′-GGCATGGACTGTGGTCATGAG-5′ (reverse). The qPCR program was as follows: (1) reverse transcription at 42°C for 30 min; (2) predenaturation at 94°C for 2 min; and (3) 35 cycles of 95°C for 20 s, 56°C for 20 s, and 72°C for 30 s [21].

### Western blot (WB)

Ovarian cancer and paracancerous fresh tissues were obtained from three of the above 102 ovarian cancer cases. Total protein was extracted from fresh ovarian cancer tissue and adjacent noncancerous tissues and quantitatively analyzed using the bicinchoninic acid assay (BCA) kit (Beyotime Biotechnology, Shanghai, China). The proteins were then subjected to sodium dodecyl sulfate polyacrylamide gel electrophoresis (SDS-PAGE) and transferred to a membrane (nitrocellulose filter membrane, NC) for immunoblotting. The membranes were then incubated with a primary SPAG5 antibody (SPAG5, 1:500, HPA022008, Sigma-Aldrich, St. Louis, MO, U.S.A.) in 5% nonfat milk, followed by a horseradish peroxidase (HRP)-conjugated anti-rabbit secondary antibody. An electrochemiluminescence (ECL) detection reagent was used to reveal results [19].

### Tissue microarray preparation and immunohistochemical analysis

Ovarian cancer tissues and paracancerous tissues from 102 ovarian cancer cases were used to create tissue microarrays. Paraffin-embedded tissues were used to prepare each specimen; 1.5-mm-diameter formalin-fixed, paraffin-embedded tissue cores were used to construct a block, and then 4-µm sections were cut from the block. The 4-µm sections were used to create the array. IHC was performed as previously described [19,20]. The SPAG5 monoclonal antibody was purchased from Sigma-Aldrich, St. Louis, MO, U.S.A. (SPAG5, 1:200, HPA022008). The secondary antibody was a horseradish peroxidase-conjugated antibody (Dako Cytomation, Carpinteria, CA, U.S.A.). The IHC results were observed by two senior pathologists. The results were evaluated according to previous studies [19,20,22] as follows: 0, negative; 1, weak positive; 2, moderate positive; and 3, strong positive. The percentage of positive cells was calculated as follows: 1, 0–10%; 2, 11–50%; 3, 51–80%; and 4, 81–100%. A sum of the scores of the above two parameters greater than or equal to 4 indicates high levels of expression, whereas a score less than 4 indicated low levels of expression.

### Statistical analysis

All statistical analyses were performed using the STATA 14.0 statistical software (Stata Corporation, College Station, TX, U.S.A.). The Wilcoxon test was used to compare SPAG5 expression in the ovarian cancer and paracancerous tissues. The chi-square test was performed to evaluate the correlation between SPAG5 expression and the clinicopathological parameters. Kaplan–Meier analysis (log-rank test) was used to assess the prognostic value. Univariate and multivariate survival analyses were performed using the Cox proportional hazard model. A difference was considered statistically significant when the *P* value was < 0.05.

## Results

### *SPAG5* expression in the GEO database and its relationship with the clinical characteristics of patients with ovarian cancer

**Figure 1:**
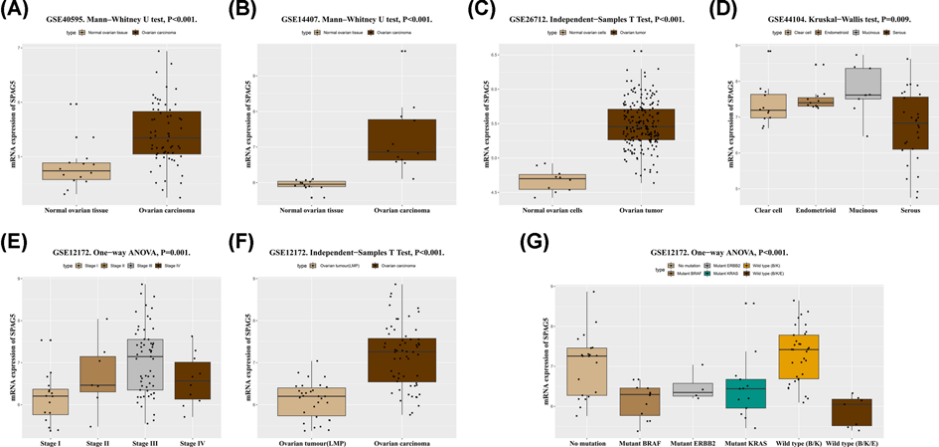
The relationship between *SPAG5* mRNA expression and clinical characteristics in the GEO database The differences in *SPAG5* mRNA expression between normal ovarian tissues and ovarian cancer tissues in the GSE40595 (**A**) and GSE14407 (**B**) datasets (all *P* < 0.001). The difference in *SPAG5* mRNA expression between normal ovarian cells and ovarian tumors in the GSE26712 dataset (**C**) (*P* < 0.001). The differences in *SPAG5* mRNA expression between various histological types in the GSE44104 dataset (**D**) (*P* = 0.009). In the GSE12172 dataset, a significant difference in *SPAG5* mRNA expression was found between different TNM stages (**E**) (*P* = 0.001). *SPAG5* mRNA expression was significantly lower in ovarian tumors with LMP than that in malignant ovarian cancers (**F**) (*P* < 0.001). *SPAG5* mRNA expression in various wild-type, mutant, and nonmutant ovarian cancers (**G**) (*P* < 0.001). Note: K: KRAS; B: BRAF; E: ERBB2.

In the GSE40595 and GSE14407 datasets, *SPAG5* mRNA expression was significantly higher in ovarian cancer tissues than that in normal ovarian tissues (both *P* < 0.001, Figure 1A,B). The GSE26712 dataset also indicated that *SPAG5* mRNA expression was significantly higher in ovarian tumors than that in normal ovarian cells (*P* < 0.001, Figure 1C). In addition, analysis of the GSE44104 dataset revealed significant differences in the high expression of *SPAG5* mRNA between distinct histologic types (*P* = 0.009, Figure 1D). By exploring the GSE12172 dataset, we found that *SPAG5* mRNA expression was significantly different between different TNM stages (*P* = 0.001, Figure 1E). Interestingly, *SPAG5* mRNA expression was decreased in stage IV tumors. Since only 10 stage IV ovarian cancer specimens were available, the sample size should be increased for further investigation. Similarly, in the GSE44104 dataset, *SPAG5* mRNA expression was higher in malignant ovarian cancer than that in ovarian tumors with low malignant potential (LMP) (*P* < 0.001, Figure 1F). As shown in Figure 1G, the expression levels of *SPAG5* mRNA were significantly different between wild-types and mutant ovarian cancers (*P* < 0.001). One-way analysis of variance (ANOVA) with least significant difference (LSD) post hoc correction was performed, and the results showed that among ovarian cancers, wild-type (*V-Raf Murine Sarcoma Viral Oncogene Homolog B1 (BRAF)/Kirsten Rat Sarcoma Viral Oncogene Homologue (KRAS)*) ovarian cancer had the highest *SPAG5* mRNA expression. *SPAG5* mRNA expression in wild-type (*BRAF/KRAS*) ovarian cancer was significantly different from that in mutant *BRAF* ovarian cancer (*P* < 0.001), mutant *KRAS* ovarian cancer (*P* < 0.001), mutant *V-Erb-B2 Avian Erythroblastic Leukemia Viral Oncogene Homolog* (*ERBB)2* ovarian cancer (*P* = 0.027), and wild-type (*BRAF/KRAS/ERBB2*) ovarian cancer (*P* < 0.001). *SPAG5* mRNA expression was lowest in wild-type (*BRAF/KRAS/ERBB2*) ovarian tumors, which differed from nonmutant tumors (*P* < 0.001) and wild-type (*BRAF/KRAS*) tumors (*P* < 0.001).

### High expression of SPAG5 in ovarian cancer

**Figure 2:**
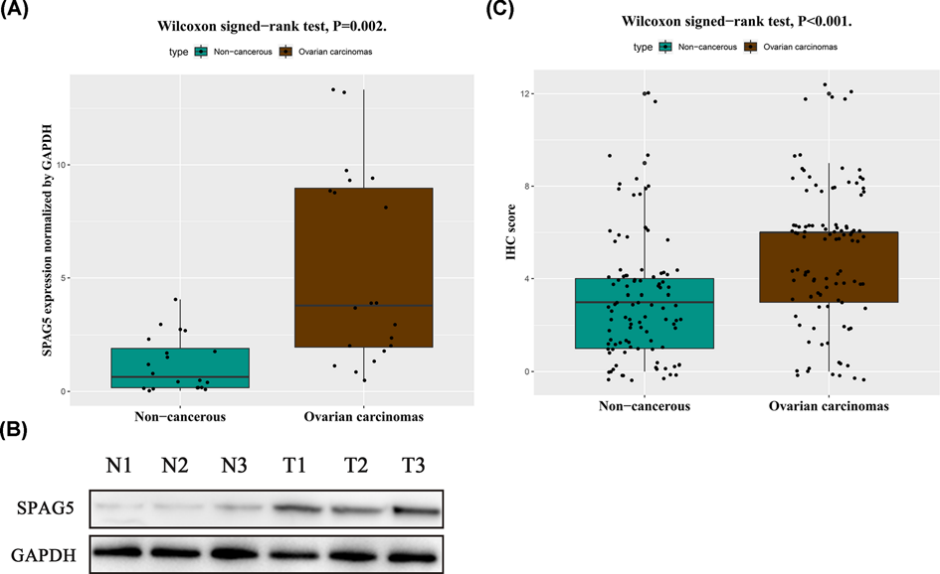
SPAG5 mRNA and protein expression in ovarian cancer tissues and matched noncancerous tissues One-step quantitative real-time polymerase chain reaction (qPCR), Western blotting (WB), and immunohistochemistry (IHC) were performed to evaluate SPAG5 mRNA and protein expression in ovarian cancer tissues and matched noncancerous tissues. *SPAG5* mRNA expression in ovarian cancer tissues was significantly higher than that in matched tumor-adjacent noncancerous tissues (*P* = 0.002) when normalized to the internal control GAPDH (**A**). SPAG5 protein expression in ovarian cancer tissues was higher than that in matched tumor-adjacent noncancerous tissues by WB (**B**) and IHC (**C**). IHC scoring data showed that SPAG5 protein expression in ovarian cancer tissues was significantly higher than that in matched tumor-adjacent noncancerous tissues (*P* < 0.001).

**Figure 3:**
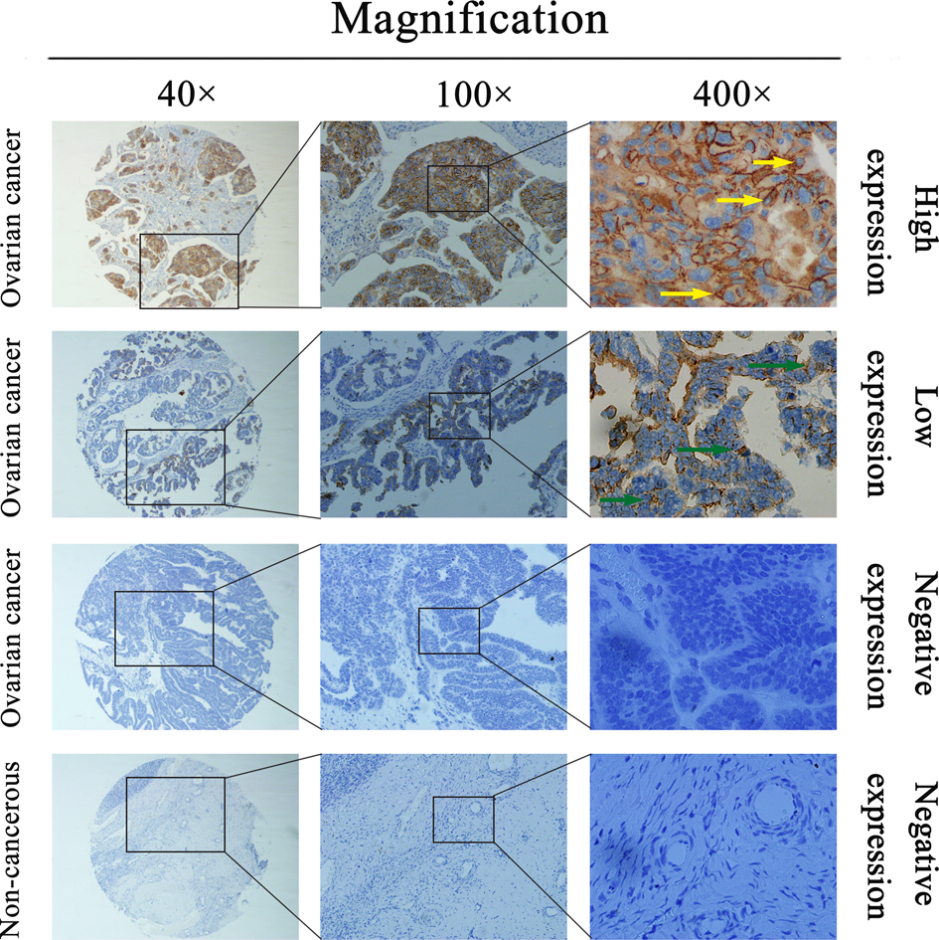
Representative images of SPAG5 protein expression in ovarian cancer and corresponding noncancerous tissues Representative images of SPAG5 protein expression in ovarian cancer and corresponding noncancerous tissues. Positive staining is evident in the cancer cell cytoplasm and membrane.

To study *SPAG5* mRNA levels in ovarian cancer patients, qRT-PCR was performed on cancerous and corresponding paracancerous tissues from 20 ovarian cancer cases. The results showed that compared with the endogenous control gene GAPDH, *SPAG5* expression levels in ovarian cancer tissues and paracancerous tissues were 5.36 ± 4.25 and 1.20 ± 1.21 (*P* = 0.002), respectively (Figure 2A). *SPAG5* mRNA expression in ovarian cancer tissues was approximately 4.5-fold that in paracancerous tissues. SPAG5 protein expression levels in ovarian cancer and paracancerous tissues was detected using WB and IHC. Consistent with the qPCR results, WB detection showed that SPAG5 expression in ovarian cancer tissues was significantly higher than that in paracancerous tissues (Figure 2B). The IHC assay showed that ovarian cancer tissues from 56 of 102 cases exhibited high SPAG5 expression and that paracancerous tissues from 42 cases had high SPAG5 expression; the difference was significant (*P* < 0.001, Figure 2C). SPAG5-positive staining was mainly located in the cytoplasm and cell membrane. Typical IHC staining for SPAG5 expression in ovarian cancer cells is shown in Figure 3. The above data indicated that SPAG5 was highly expressed in ovarian cancer tissues.

### Correlation of SPAG5 expression with the clinicopathological features of ovarian cancer

The association between high SPAG5 expression in ovarian cancer and patient clinicopathological features is shown in Table 1. High SPAG5 expression was correlated with histological type (*P* = 0.009); lymph node metastasis (*P* = 0.001); distant metastasis (*P* = 0.001); tumor, node, metastasis (TNM) stage (*P* = 0.001); and prognosis (*P* = 0.001). Other important clinicopathological indicators, such as age, tumor size and location, and tumor grade were not correlated with SPAG5 expression.

**Table 1:** Relationship between SPAG5 overexpression and the clinicopathological characteristics of ovarian carcinomas patients

Groups	No.	SPAG5		χ^2^	*P* value
		+	%		
Total	102	56	54.9		
Age (years)					
≥60	28	16	57.1	0.08	0.780
<60	74	40	54.1		
Tumor size (cm)					
>5	86	49	57.0	0.95	0.329
≤5	16	7	43.8		
Histological type					
Serous	67	42	62.7	11.53	0.009*
Mucinous	17	8	47.1		
Endometroid	13	2	15.4		
Clear cell	5	4	44.4		
Pathological grade					
Grade 1	16	5	31.3	4.34	0.114
Grade 2	21	12	57.1		
Grade 3	65	39	60.0		
Lymph node metastasis					
Positive	27	25	92.6	21.07	0.001*
Negative		75	31	41.3	
Distant metastasis					
Positive	21	18	85.7	10.14	0.001*
Negative	81	38	46.9		
TNM stage					
Stage I	3	0	0.00	17.62	0.001*
Stage II	21	6	28.6		
Stage III	57	32	56.1		
Stage IV	21	18	85.7		
Survival					
Yes	43	11	25.6	25.81	0.001*
No	59	45	76.3		

* *P* < 0.05

### The effect of SPAG5 expression on ovarian cancer patient survival

**Figure 4:**
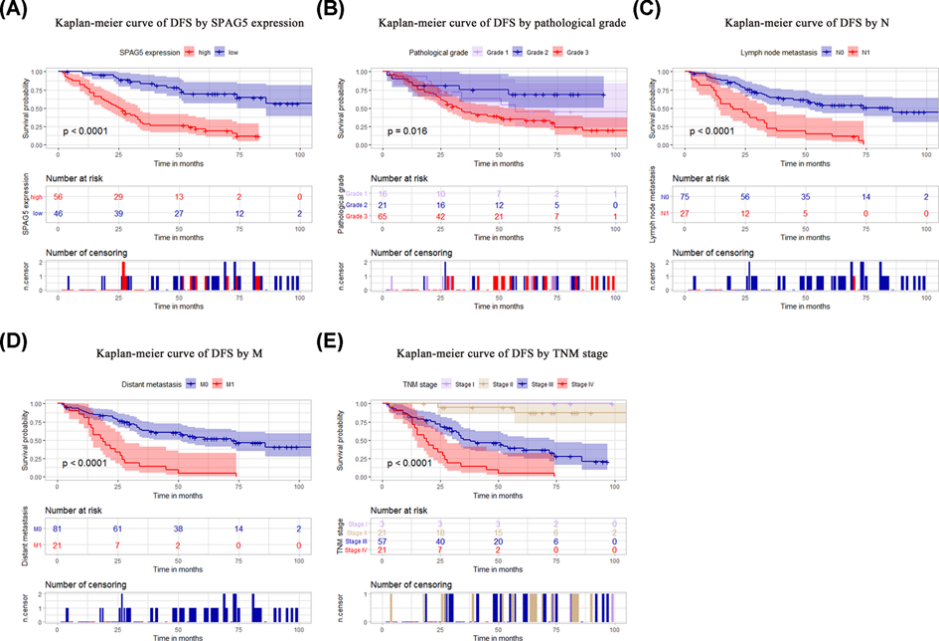
The prognostic value of SPAG5 expression for the DFS of ovarian cancer The Kaplan–Meier method was used to analyze ovarian cancer patient survival. Patients with high SPAG5 expression, an advanced pathological grade, or an N1, M1, or advanced TNM stage of disease exhibited worse DFS rates (**A**–**E**). The *P* value was determined using the log-rank test.

**Figure 5:**
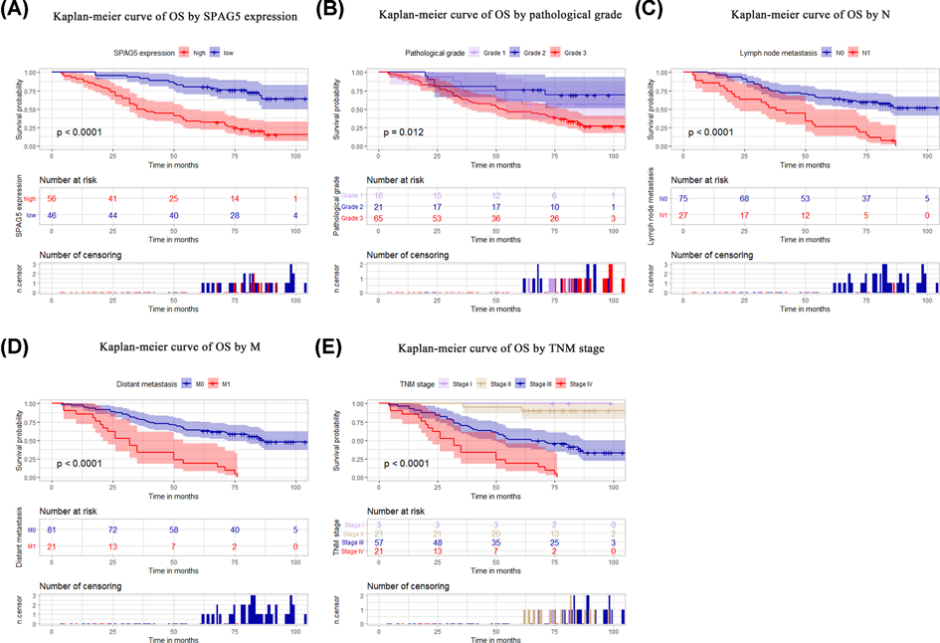
The prognostic value of SPAG5 expression for the OS of ovarian cancer Patients with high SPAG5 expression, an advanced pathological grade, or an N1, M1, or advanced TNM stage of disease exhibited worse OS rates (**A**–**E**). The *P* value was determined using the log-rank test.

The Kaplan–Meier curve indicated that the disease-free survival (DFS) rate was lower among patients with high SPAG5 expression (*P* < 0.001), an advanced pathological grade (*P* = 0.016), or an N1 (*P* < 0.001), M1 (*P* < 0.001), or advanced TNM (*P* < 0.001) stage of disease (Figure 4A–E). The curve also indicated that overall survival (OS) was lower among patients with high SPAG5 expression (*P* < 0.001), an advanced pathological grade (*P* = 0.012), or an N1 (*P* < 0.001), M1 (*P* < 0.001), or late TNM (*P* < 0.001) stage of disease (Figure 5A–E). Univariate analysis revealed that high SPAG5 expression (*P* = 0.001), tumor pathological grade (*P* = 0.031), lymph node metastasis (*P* = 0.001), distant metastasis (*P* = 0.001), and TNM staging (*P* = 0.001) were correlated with DFS in the 102 patients with ovarian cancer (Table 2). High SPAG5 expression (*P* = 0.001), tumor pathological grade (*P* = 0.022), lymph node metastasis (*P* = 0.001), distant metastasis (*P* = 0.001), and TNM stage (*P* = 0.001) were also correlated with the OS of the 102 ovarian cancer patients (Table 3). Further multivariate analysis showed that SPAG5 expression (*P* = 0.001) and TNM staging (*P* = 0.002) could be used as independent prognostic factors for DFS (Table 2). SPAG5 expression (*P* = 0.001) and TNM staging (*P* = 0.005) were also independent prognostic factors for OS (Table 3; Figure 5).

**Table 2:** Univariate and multivariate analyses on the disease-free survival of 102 cases of ovarian cancer

	Univariate analysis			Multivariate analysis		
	HR	*P* > |*z*|	95% CI	HR	*P* > |*z*|	95% CI
SPAG5 expression						
High versus Low	5.06	0.001*	2.705–9.458	3.57	0.001*	1.782–7.158
Age (years)						
≥60 versus <60	1.26	0.417	0.719–2.216			
Tumour size (cm)						
>5 versus ≤5	2.23	0.064	0.955–5.191			
Histological type						
Serous versus mucinous versus Endometroid versus clear cell	1.22	0.250	0.870–1.704			
Pathological grade						
Grade 1 and 2 versus Grade 3	0.63	0.031*	0.417–0.958	0.98	0.936	0.645–1.497
Lymph node metastasis						
Positive versus negative	3.77	0.001*	2.225–6.379	1.04	0.909	0.550–1.958
Distant metastasis						
Positive versus negative	4.38	0.001*	2.507–7.666	4.42	0.070	0.886–22.099
TNM stage						
Stage I versus Stage II versus Stage III versus Stage Stage IV	0.26	0.001*	0.167–0.392	0.11	0.002*	0.025–0.447

* *P* < 0.05

**Table 3:** Univariate and multivariate analyses on the overall survival of 102 cases of ovarian cancer

	Univariate analysis			Multivariate analysis		
	HR	*P* > |*z*|	95% CI	HR	*P* > |*z*|	95% CI
SPAG5 expression						
High versus low	4.61	0.001*	2.511–8.454	3.05	0.001*	1.547–6.031
Age (years)						
≥60 versus <60	1.26	0.412	0.723–2.204			
Tumour size (cm)						
>5 versus ≤5	2.30	0.055	0.984–5.372			
Histological type						
Serous versus mucinous versus Endometroid versus clear cell	1.28	0.140	0.921–1.784			
Pathological grade						
Grade 1 and 2 versus Grade 3	0.62	0.022*	0.408–0.933	0.94	0.785	0.622–1.431
Lymph node metastasis						
Positive versus negative	3.62	0.001*	2.150–6.110	1.05	0.877	0.549–2.022
Distant metastasis						
Positive versus negative	4.56	0.001*	2.604–7.987	3.37	0.138	0.677–16.761
TNM stage						
Stage I versus Stage II versus Stage III versus Stage Stage IV	0.25	0.001*	0.163–0.392	0.13	0.005*	0.031–0.543

* *P* < 0.05

## Discussion

SPAG5 is a spindle-binding protein that regulates the progression of mitosis. Recent studies have found that SPAG5 is closely related to tumor occurrence and development. In cervical cancer [14], breast cancer [15,16], bladder cancer [17], HCC [18], lung cancer [23], prostate cancer [24] and other malignancies, SPAG5 is highly expressed in tumor tissues, indicating a poor prognosis. SPAG5 promotes proliferation and suppresses apoptosis in bladder urothelial carcinoma at least partially via up-regulating Wnt3 by activating the AKT/mTOR signaling pathway [17]. SPAG5 might be an independent prognostic and predictive biomarker that may have clinical utility as a biomarker for combination cytotoxic chemotherapy sensitivity, especially in estrogen receptor-negative breast cancer [16]. In human non-small cell lung cancer (NSCLC), miR-1179 inhibited the growth and invasion of NSCLC cells by targeting SPAG5 and inhibiting Akt, highlighting the importance of the miR-1179/SPAG5/Akt axis in the progression of NSCLC [25]. SPAG5 expression was gradually increased during prostate cancer progression and its level was significantly associated with lymph node metastasis, clinical stage, Gleason score, and biochemical recurrence; moreover, miR-539 can inhibit prostate cancer progression by directly targeting SPAG5 [24]. Recent studies have shown that SPAG5 promotes HCC progression by down-regulating SCARA5 by modifying β-catenin degradation [26]. SPAG5 serves as a promising prognostic factor in HCC and functions as an oncogene via the CEP55-mediated PI3K/AKT pathway; thus, the miR-363-3p/SPAG5/CEP55 axis may represent a potential therapeutic target for HCC clinical intervention [27]. The above results indicate that SPAG5 may be an oncogene that promotes tumor progression, invasion, and metastasis. However, the role of SPAG5 in ovarian cancer has rarely been reported. Whether SPAG5 can be used as a new marker for the diagnosis and prognosis of ovarian cancer was the focus of the present study.

qPCR, WB, and IHC analyses were used to study the mRNA and protein expression levels of SPAG5 in ovarian cancer. *SPAG5* mRNA expression was significantly higher in ovarian cancer tissues than in matched noncancerous tissues. The same results were confirmed by WB and IHC. Therefore, the carcinogenic function of *SPAG5* in ovarian cancer has been confirmed. Our study also found that SPAG5 is correlated with the clinicopathological features of ovarian cancer, including histological type, lymph node and distant metastasis, TNM staging, and prognosis. Our results are consistent with previous studies on SPAG5 in other tumors [14–18,23,24].

Univariate analysis showed that high SPAG5 expression, pathological grade, lymph node metastasis, distant metastasis, and TNM staging were correlated with DFS and long-term survival of patients with ovarian cancer. Multivariate survival analysis showed that high SPAG5 expression and TNM staging could be used as individual prognostic factors for ovarian cancer. The Kaplan–Meier curve also showed that patients with high SPAG5 expression and TNM staging had shorter survival. This finding is consistent with the results from recent studies that also found a role for SPAG5 in the prognosis of related tumors [14–18,23,24].

We believe that the study of SPAG5 in tumors requires further in-depth studies, as there are likely many factors that influence SPAG5 in different tumor studies, such as tumor typing, antibody sources, and quality control. Our study also lacks the support of a large-scale sample size, and subsequent studies on *in vivo* and *in vitro* models to investigate the biological role of SPAG5 in ovarian cancer invasion and metastasis and the regulation of related signaling pathways are underway.

In summary, the present study is the first full manuscript to report the relationship between SPAG5 expression and clinicopathological features in ovarian cancer, especially its prognosis in details. Our results showed that SPAG5 could be used as a potential tumor marker for ovarian cancer. Further studies including studies on larger sample sizes and those examining the biological roles of SPAG5 in ovarian cancer invasion and metastasis are necessary.

## Abbreviations

95% CI: 95% confidence interval
AJCC: the American Joint Committee on Cancer
BCA: bicinchoninic acid assay
BRAF: V-Raf Murine Sarcoma Viral Oncogene Homolog B1
CA125: cancer antigen 125
DFS: disease-free survival
ECL: electrochemiluminescence
ERBB: V-Erb-B2 Avian Erythroblastic Leukemia Viral Oncogene Homolog
GEO: Gene Expression Omnibus
HCC: hepatocellular carcinoma
HE4: human epididymis protein 4
HR: hazard ratio
IHC: immunohistochemistry
KRAS: Kirsten Rat Sarcoma Viral Oncogene Homologue
mRNA: messenger RNA
NC: nitrocellulose filter membrane
NSCLC: non-small cell lung cancer
OS: overall survival
qPCR: one-step quantitative real-time polymerase chain reaction
SDS-PAGE: sodium dodecyl sulfate polyacrylamide gel electrophoresis
SPAG5: sperm-associated antigen 5
TMA: tissue microarray
TNM: tumor node metastasis
WB: Western blot
